# The Blind Call for More Research

**DOI:** 10.1111/jep.70155

**Published:** 2025-06-10

**Authors:** Jacob Alexander de Ru

**Affiliations:** ^1^ Department of Otorhinolaryngology – Head Neck Surgery Central Military Hospital Utrecht Lundlaan 1 Utrecht The Netherlands

“*Fortunately I believe we have not yet gone so far as our friends in the USA where, I am told, some editors of journals will return an article because tests of significance have not been applied,*” was written more than half a century ago in one of the most influential papers in medical history [1].

Unfortunately, recently an editor indeed returned a manuscript stating: “with that said, I urge you to conduct a more formal study of the usefulness of this maneuver in anesthetized patients and report your result.” Though at first glance this might not be a too strange remark, the ‘maneuver’ in this case is just a simple and harmless head‐turn.

A can't oxygenate, can't ventilate, can't intubate situation means that a patient cannot breathe because he/she is under anesthesia, and doesn't get oxygen because the anesthesiologist cannot secure the airway. A can't oxygenate, can't ventilate, can't intubate situation may result in a life‐threatening situation thus, a movement as simple as turning the patient's head can be worth every penny.

During Drug Induced Sleep Endoscopy (DISE), a diagnostic procedure used in case of obstructive sleep apnea syndrome (OSAS), we noticed that turning the head while the patient is under anesthesia can open the airway if it is obstructed by the base of the tongue. The head‐turn is a routine part of the procedure as it might be an indicator of the effect of positional therapy for OSAS.

The ‘instructions for authors’ of the specific journal we had in mind states: ‘ “Freestanding” Letters to the Editor also may discuss matters of general interest to anesthesiologists, without specific linkage to recently published articles,’ and, “Letters to the Editor should be brief (250 to 750 words).”

Therefore, we wrote a 250 words letter to the editor with the advice for colleagues to try, based on our experience during DISE, a simple head‐turn for non‐traumatic cases of can't oxygenate, can't ventilate, can't intubate situation [2].

Even the editor found our “idea interesting and perhaps useful to many anesthesiologists.”

Unfortunately, since ‘evidence based’ became the magic words in medicine, the focus seems to have shifted from clear and logical thinking to an adopted creed for more and more research. However, in many cases we do not need more research to improve healthcare or to learn easy tips and tricks. To quote Hill concerning tests of significance: “*yet there are innumerable situations in which they are totally unnecessary – because the difference is grotesquely obvious, because it is negligible, or because, whether it be formally significant or not, it is too small to be of any practical importance* [1].”

Chalmers, Sackett and Silagy wrote: “*when care has such striking effects, (…) carefully controlled research is seldom necessary to identify whether the prescriptions and proscriptions of doctors and other health professionals are more likely to do good than harm*” [3]. Even Cochrane stated that formal research is not the only way for “*therapies with no backing from RCTs, which are justified by their immediate and obvious effect*” [4].

And everyone has read the parody in the BMJ Christmas 2003 edition concerning parachute use in case of jumping out of a plane [5]. But, though many colleagues have quite a laugh from reading that brilliant paper, they are still unable to translate its conclusion into daily medical practice [6].

Like the parachute, this ‘maneuver’ is not a maneuver in need of a trial. It is too simple for not trying: you turn the head and you'll either notice that ventilation became possible or it didn't help you at all (and you turn the head back).

Conducting a more formal study on this subject would be an unworkable situation for a couple of reasons: 1) it would be a waste of time and money; 2) it would be quite impossible to perform an RCT; 3) the outcome would probably be irrelevant.

Ad 1) Clinical research is subject to increasing regulation by research ethics committees and research and development offices [7]. So, performing a trial, costs an enormous amount of time and money from the research team. It would cost me personally the time and money to study and take the obligated exam to be allowed to do research on humans. Furthermore, I would have to write a protocol that needs to be approved by our Medical Ethical Committee.

Compliance with current regulations is so complex, time‐consuming and therefore prohibitively expensive, that research will become unaffordable except by industry [7]. As stated by Warlow: the resulting bureaucracy, expense and confusion are putting insuperable hurdles in the way of clinical research and clinical care is compromised [7]. Unfortunately, delays can cost lives [7].

Performing formal research in this case doesn't weigh up against the costs (both the time and the money).

Ad 2) If a simple letter for educational purpose is not good enough, and we are urged to conduct a formal study, the editor might be thinking about an RCT. However, when do we ask for informed consent for the trial? When we meet the situation of can't oxygenate?

At what moment in time are we supposed to randomize the patient, when he or she is at 80% oxygenation?

Of course, one could argue that we better ask for a patient's signature before the operation. But, if a can't oxygenate, can't ventilate, can't intubate situation happens 1 in 20,000 cases, we just need 1,600,000 participants for a trial with 40 patients in both groups.

Even the apparent solution of asking patients during routine clinical care if their data can be used for research is not as simple as it may seem [3]. So just waiver the consent?

Furthermore, templates for providing patients with information can result in inappropriate, lengthy and unclear leaflets [7].

3) If 80% of a cohort would do better, it is a very successful procedure. If the treatment group is significant 20% better than the control group, it is a successful procedure. If it only works in 1 out of twenty patients it is still worth a try for a patient without oxygen.

Only one specific outcome would be meaningful: the maneuver is unsuccessful in every case. In all other cases the outcome is irrelevant if you compare the situation to the maneuvre. (We probably wouldn't have written the letter if we didn't have some positive experience with it).

In case doubt still exists on the effectiveness of a maneuver like a head‐turn, I would suggest that a colleague who wants to try the maneuver, calculate a rate ratio ‐ as described by Glasziou et al. ‐ for him or herself, and see if the airway that was obstructed opens up during the clinical situation in need of it [8].

In my opinion, confirmation of the effectiveness, the striking effect, of the maneuver by other authors is more useful than a formal study.

To conclude, I like to end with a citation from the same masterpiece I started with, because performing formal research on the topic of a head‐turn is probably what Hill had in mind when he wrote: “*yet too often I suspect we waste a deal of time, we grasp the shadow and lose the substance, we weaken our capacity to interpret data and to take reasonable decisions whatever the value of P.*” [1].

## Mesh

Evidence‐Based Medicine, logic.

## Conflicts of Interest

The author declares no conflicts of interest.

## Data Availability

Data sharing not applicable to this article as no datasets were generated or analyzed during the current study.
